# Jellyfish as Food: A Narrative Review

**DOI:** 10.3390/foods11182773

**Published:** 2022-09-08

**Authors:** António Raposo, Ibrahim Alasqah, Hani A. Alfheeaid, Zayed D. Alsharari, Hmidan A. Alturki, Dele Raheem

**Affiliations:** 1 CBIOS (Research Center for Biosciences and Health Technologies), Universidade Lusófona de Humanidades e Tecnologias, Campo Grande 376, 1749-024 Lisboa, Portugal; 2 Department of Public Health, College of Public Health and Health Informatics, Qassim University, Al Bukairiyah 52741, Saudi Arabia; 3 Department of Food Science and Human Nutrition, College of Agriculture and Veterinary Medicine, Qassim University, Buraydah 51452, Saudi Arabia; 4 Department of Clinical Nutrition, Faculty of Applied Medical Sciences, Tabuk University, P.O. Box 741, Tabuk 71491, Saudi Arabia; 5 General Directorate for Funds & Grants. King Abdulaziz City for Science & Technology, P.O. Box 6086, Riyadh 11442, Saudi Arabia; 6 Northern Institute for Environmental and Minority Law (NIEM), Arctic Centre, University of Lapland, 96101 Rovaniemi, Finland

**Keywords:** jellyfish, sustainability, health, food security, food safety, nutrition, sensory evaluation, food technology

## Abstract

Studies toward a sustainable future conducted by international organizations uniformly agree about having to change some of our present consumer behaviors. Regarding food, suggestions include eating locally farmed, less industrialized and renewable food to promote health and circularity, and limiting waste. Jellyfish are frequently sorted and discarded after being caught with fish in fishing nets and gear. In contrast, we propose utilizing this by-catch as food. This review discusses the economic value and sustainability of jellyfish, the technologies used to prepare them for human consumption, their nutritional profile and health impacts and, finally, consumer acceptability and sensory evaluation of jellyfish food products. This discussion is critical for promoting jellyfish as an important aquatic resource to support blue and circular economies.

## 1. Introduction

Changing many current consumer behaviors has been universally acknowledged in studies toward a sustainable future that have been conducted by international organizations. Consuming locally grown, minimally industrialized and renewable foods is encouraged [1,2,3].

From 1988 to 1999, estimates of the average annual jellyfish capture worldwide exceeded 321,000 metric tons [4]. In 2016, the Food and Agriculture Organization (FAO) reported that jellyfish catches were globally increasing, but provided no specific statistics [5]. The approximate caught amounts of octopus and cuttlefish were respectively 350,000 and 300,000 [5]. Although the world locations where they are consumed do not appear to be the same, these findings imply that jellyfish consumption is like that of octopus and cuttlefish worldwide. One of Georgia’s top three fisheries currently fishes jellyfish to be sold to Asia for food [6,7,8].

The phylum Cnidaria’s Scyphozoa class is where jellyfish are classified. These animals are thought to come in 200 different species. Only the mature Rhizostomae, also known as medusas, are categorized into four orders that are assumed edible [9]. Umbrella, tentacles and oral arms make up the main medusan body. Removing the edges and reproductive and digestive tracts leaves the umbrella, which is the component that can be eaten. Jellyfish tentacles and oral arms contain toxins that can be dangerous to humans depending on the species [10]. 

Since ancient times, the Chinese culture has consumed jellyfish in the medusa stage. The practice of eating cooked umbrella in salads has extended to other Asian nations such as Malaysia, Thailand and Japan. Europe is beginning to see this possibility [11,12,13].

Eating jellyfish is seen as having health benefits in Chinese popular culture, and both its flavor and texture are acclaimed [14]. According to scientific studies, jellyfish that are edible possess a kind of collagen that can be hydrolyzed enzymatically to produce distinctive peptides with antihypertensive action [15,16]. Jellyfish mucin, known as qniumucin [17], is also a crucial element in the formulation to treat joint diseases [18].

Techniques to produce food in the future, made possible by scientific research and technology, will have to bear in mind environmental sustainability, waste reduction and the ability to eat novel food to meet the ever-growing need [19,20]. Of these food options, jellyfish can be a viable substitute for traditional proteins with minimal intake of carbohydrates and saturated fats. Their consumption may also help to prevent slow fish biodiversity loss [21].

Using Western jellyfish as potential food is enabled by the European novel food regulations (Regulation 2015/2283), and by the identification and biochemical characterization of the bioactive properties of certain jellyfish species from the Mediterranean. The latter are included on the so-called “novel food” list. However, it is advisable to assess evidence for their innocuousness, i.e., any likely allergic, chemical, physical and microbiological concerns associated with humans consuming them, before these products are commercialized and their large-scale consumption in Europe [22].

Based on these premises, this review aims to study: the sustainability and economic relevance of jellyfish; their food processing technologies for human consumption; the potential risks associated with eating jellyfish and their nutritional profile and health impacts; and consumer acceptance and the sensory analysis of jellyfish food products.

## 2. Sustainability and Economic Relevance

The relevance of the jellyfish value chain for sustainability is twofold, and involves positive and negative aspects. First, utilization of jellyfish will contribute to develop the blue economy and to foster sustainability. There is an interest in using other food resources to cope with feeding our growing population. This requires developing mass culture systems to maintain the rising demand for edible jellyfish and their by-products [23,24]. Jellyfish are considered a valuable bioresource with applications in cosmeceutical, nutraceutical, pharmaceutical and, generally, in biotechnological applications [25,26,27]. Second, there is the direct negative impact of jellyfish on farmed fish stock mortality. Fish gill tissue can be damaged by physical impacts from jellyfish exposure, because jellyfish are ubiquitous in marine environments, which could have another negative impact for aquaculture. Cnidarian species’ surface microbiome is specialized and differs from environmental bacterial populations by hosting a wide-ranging population of bacteria [28]. It has been established that complicated gill disorders are a very serious cause of marine-farmed salmon death in Ireland with average 12% losses per year [29,30,31].

Normally, jellyfish venom injection and nematocyst discharge result in cell toxicity, histopathology and a local inflammatory response [30,32,33]. Prolonged nematocyst discharge in fish tissue could imply not only secondary bacterial infections, but also associated systemic reactions that include respiratory and osmoregulatory distress, alterations to behaviors and even mortality [33,34,35]. Some jellyfish species are capable of acting as either vectors for *Tenacibaculum maritimum*, which is the causative agent of tenacibaculosis [36], or potential reservoirs of the causal amoebic gill disease agent, namely *Neoparamoeba perurans* [37]. These two main pathogens can affect fish farming and substantially globally, making the impact of jellyfish injuries worse [38,39,40,41]. 

Economic loss caused by jellyfish outbreaks has had a significant influence on aquaculture [42,43,44]. Some scientific works include distinct events in both the Northern Europe and the Mediterranean Seas to have resulted in economic loss. Scottish and Irish aquaculture have been repeatedly hit by extremely heavy economic loss (as much as USD 1.3 million) because of recurrent *P. noctiluca* invasions causing substantial salmon mortality rates [44,45].

Therefore, ecosystem-based (E-based) fisheries management that integrates ecosystem components, including humans, into the decision-making process is required so that managers can balance trade-offs and better determine what management decisions are required [46]. They must be fully integrated for impacts to be meaningful, which will increase predictability as a result of the improved coordination of processes and have more compatible and accessible scientific data [47]. 

E-based fishery management has been suggested to safeguard a comprehensive approach for the sustainability of jellyfish fisheries that will be positive. To ensure environmental sustainability, certain issues like habitat integrity, monitoring, by-catches, seasonality and the life cycle of products have to be taken into account so they can be managed [13]. This implies taking integrated approaches to include state-of-the-art fishing management strategies within ecological boundaries by integrating fishing along with other human actions, and preserving ecosystem stability and biodiversity with the necessary processes to ensure the stability of ecosystems and/or their services [48].

According to the Food and Agriculture Organization (FAO), the total world capture production of edible jellyfish from 2015 to 2018 was estimated as 300,000 tons/year. [49]. Global edible jellyfish production of live, fresh, dried, frozen, chilled, brined or salted jellyfish products was estimated at approximately 10,000–17,000 tons/year between 2011 and 2015, and continues to grow. This represents an increasing business trend of USD 20–100 million [50,51]. Hence, it is economically relevant. Commercially, the value of the jellyfish products produced to be consumed by humans varies according to the product type and species, ranging from 2000 to 10,000 USD/ton, and today’s market has a stable value of around 2500 USD/ton [7,51,52]. The majority of jellyfish products/by-products are almost exclusively exported to China, Japan and South Korea, where market demand remains at its highest level to date [7,53]. Processing jellyfish as food for humans as described in the next section often involves employing huge amounts of water, which generates sustainability concerns about the water footprint and safety in water quality terms.

## 3. Food-Processing Technologies

Processing jellyfish as human food needs to be performed within hours of being harvested because jellyfish are prone to spoilage. Oral arms are removed from umbrellas and washed in large volumes of water to eliminate sand, bacteria, mucus and gonads. Both oral arms and umbrellas are processed for consumption. Next they are soaked in an NaCl/aluminum salt mixture (AlNH_4_(SO_4_)_2_12H_2_O or KAl(SO_4_)_2_12H_2_O) at a varying ratio depending on the followed method [14]. It is necessary to repeat this procedure a few times to progressively reduce the alum salt concentration. Consequently, this operation lowers water content and alters the gelatinous jellyfish tissue for it to gain the consistency expected for edible end products. Texture is generally characterized as being firm but crispy, which the Eastern market very much appreciates. Oral arm products have a lower market value compared with umbrellas [14]. 

Alum metal ions modify the mechanical–chemical properties of jellyfish tissue and have rubber-like hardening effects that is likely due to cross-linked collagen jellyfish fiber and the partial disinfection of jellyfish material [14]. According to the applied treatment and the given jellyfish species, the whole procedure can take 4–40 days [54]. Gaining a better understanding of the gelatin gel properties caused by peptide or protein contents is useful for enhancing gelatin functionality in both non-food and food applications. A recent study has demonstrated that the concentration in HCl pretreatment and extraction time strongly impact the gelling/melting temperatures, viscosity and gel strength, of jellyfish gelatin. Accomplishing the greatest gelatin gel strength is conducted by submitting jellyfish to 0.1 M HCl, and performing extraction for 12 h at 60 °C. Jellyfish gelatin has lower gel strength, viscosity, and gelling/melting temperatures than commercially produced bovine/fish gelatins [55]. 

**Figure 1:**
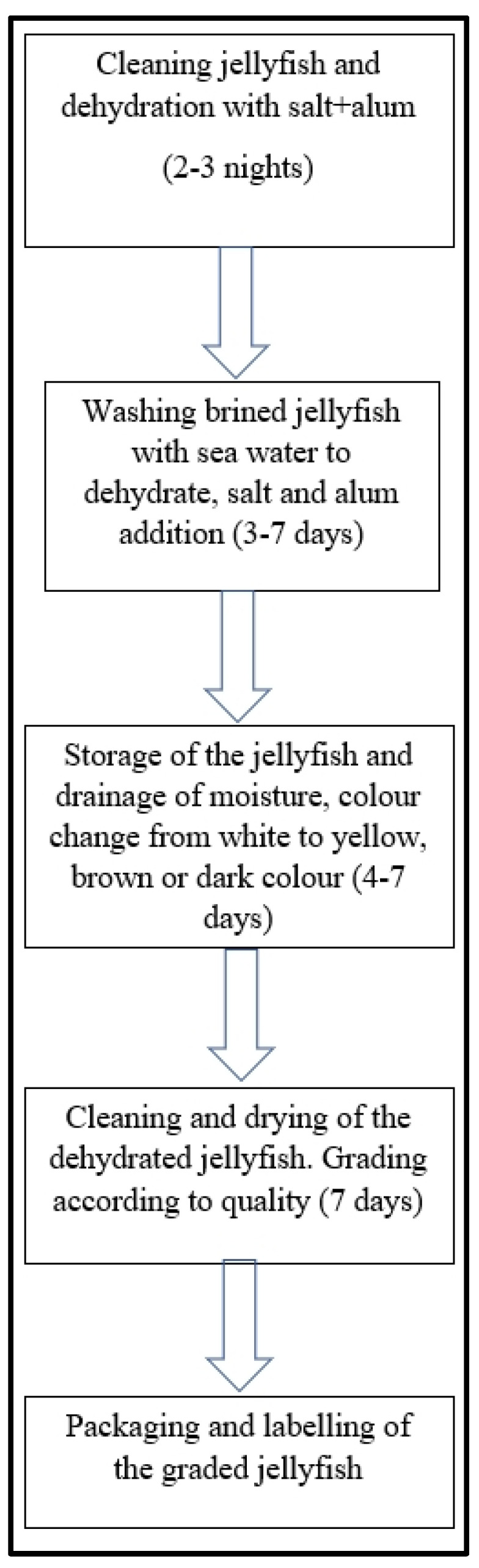
Flow chart of the steps involved in traditional jellyfish processing.

Raw jellyfish are submitted to a salt/alum (or sodium bicarbonate) mixture in the initial processing stage. A large amount of alum and salt is normally required for processing jellyfish. Used as an agent to clarify water, alum has been reported to form part of rural jellyfish processing in Sarawak, a region of Malaysia. It has neither odor nor color, but a sweetish, yet astringent, taste. It is often available as transparent, big and hard crystals [56]. Alum is popularly and widely employed as a firming agent to pickle fruit and vegetables. However, some concerns about its safety have been voiced [57,58]. Salting is the oldest food preservation technique thanks to its ability to lower food’s water content and to inhibit the growth of microorganisms [59]. The salt and alum mixture that is added to jellyfish processing as a preservative agent tastes acidic and its texture is crunchy and drier. Figure 1 illustrates the traditional method followed to process jellyfish.

The classic preparation procedure combines higher-valence and monovalent salts, which is explained by tanning analogy. Nevertheless, refined notions stemming from soft matter and polymer physics indicate novel preparation operations that employ selective solvents like ethanol. 

Figure 1 depicts the traditional jellyfish processing process in Sarawak, a rural community in Malaysia, as described by Shin et al. [56]. Cleaned jellyfish are arranged in layers. Then salt, alum and sodium bicarbonate are added for dehydration, which takes 2–3 nights. In the second stage, brined jellyfish are washed with saltwater, which leads to further dehydration and shrinking. After 3–7 days, washed jellyfish are moved to the next stage, when the base of the compartments is perforated to allow moisture to drain, which lowers jellyfish moisture content. Prolonged storage can last between 3–7 days, where changes in color from white to yellow, brown or a dark color can occur [56]. Then dehydrated jellyfish are cleaned and dried. Sand, mucus and other debris are brushed off the jellyfish prior to grading, which is based on size, color and firmness [60]. Table 1 includes a common description of the various processed jellyfish grades.

The final process stage is packaging. Graded jellyfish are packed in wooden boxes and each box weighs roughly 25–28 kg. Once boxes have been labeled with their grade, seafood distributors buy the end products.

Salted jellyfish must be prepared by desalting and rehydrating before cooking. Conventional desalting is conducted by performing many washes, and overnight soaking is quite cumbersome. Efforts are made to reduce the many washing steps with a mechanical washing machine to clean and reduce the jellyfish salt content [61]. The washing machine has a circular tank equipped with a rotating blade. A prolonged wash cycle alters the physicochemical properties of desalted jellyfish by-products [61,62]. The texture of the prepared jellyfish can be predicted during this procedure.

Jellyfish structural integrity is affected by collagen and elastin biopolymers. Jellyfish movement has been described to be equally affected by stability and elasticity, which are respectively defined by collagen and elastin [63,64], while addition of salt results in crunchy textures. Processing jellyfish without salt has been considered and solvents such as ethanol are applied. Ethanol restructures mucoproteins. This results in the formation of networks and brings about a new macroscopic structure that differs from that of living jellyfish. Once the drying stage has ended, this operation confers a crispy texture. 

Elastin helps to maintain elastic deformation, whereas collagen resists stretching. The two very different ways to prepare jellyfish as food indicate how the several polymer types of mesoglea play distinct roles in line with the followed preparation technique. Collagen plays a key role when salting jellyfish. Leaving jellyfish immersed in poor solvent gives elastin, mucopolysaccharides and mucoproteins, which confer the most prevailing effects. Jellyfish collagen is edible and may be employed to create a varied group of dry food ingredients, such as thickeners, stabilizers and collagen-peptide supplements. Ionic gels reversibly collapse and swell in good and poor solvents, respectively [65], and can inspire new jellyfish preparation strategies. It has actually been reported that jellyfish collapse in ethanol due to a faster decrease in their relative weight compared with the conventional alum-based method [66]. During this procedure, jellyfish are exposed to 96% ethanol that is allowed to evaporate over night at room temperature. This results in paper-like preserved jellyfish [66] because of the more rapidly decreasing relative weight than in the traditional alum-based operation [66]. Thus, jellyfish can be produced in 2–3 days versus the previously described traditional 1-month preparation technique.

## 4. Potential Risks Related to Eating Jellyfish

Jellyfish are a highly perishable raw material, which means that they are typically treated shortly after being collected to prevent deterioration and to preserve their organoleptic and safety properties [67]. An absence of safety concerns for human health based on currently available scientific reports is a crucial requirement to be met for the EU Commission to authorize novel food and for it to be included on the European Union list [22]. This section focuses on the main potential risks of eating jellyfish.

### 4.1. Microbiological Risks

Pathogenic microorganisms do not appear among the microbiological risk analysis results in the reviewed literature. In line with European Commission Regulations numbers 2073/2005 and 1441/2007 about food safety, the microbiological profile of a particular jellyfish, *Catostylus tagi,* was investigated by Raposo et al. [12]. The analysis centered on investigating *Listeria monocytogenes*, *Aeromonas hydrophila*, *Vibrio* spp. and *Salmonella* spp. According to the results, none of the evaluated pathogenetic markers were found, and there was no evidence for viruses and fungal biota contamination. A review of the literature demonstrates that jellyfish pose no major microbiological risk for human beings [58]. Further research must take into account metagenomic and metabolomic approaches to examine both raw and cooked jellyfish. Finding more about the total microbiota linked with jellyfish, along with quantitative/qualitative data about microbial metabolites, might be interesting to determine other sources of microbiological risks posed for human beings [67].

### 4.2. Chemical Risk

No hazardous substances such as Hg, Pb and Cd, and inorganic Sn, have been detected within detection limits (0.01 weight %) [68]. Jellyfish habitats have a significant impact on the components in jellyfish, particularly trace elements.

Other research works have revealed that the bioaccumulation process makes jellyfish particularly vulnerable to marine contaminants. A research work by Epstein et al. assessed the amount of the trace metals that the *Cassiopea maremetens* jellyfish species absorbed and retained. Metal started to quickly accumulate in jellyfish tissue from exposure to treated water within 24 h. Cu concentrated at 2.627 ± 0.031 µg/g, which suggests an almost 18.1% increase of ambient concentrations for high-nutrient conditions (analysis of variance: F1,16 = 7.436, *p* = 0.015) [69].

The *Rhizostoma pulmo* jellyfish’s potential for bioaccumulating trace elements in a Mediterranean coastal lagoon in Southeastern Spain was examined in another study [70]. In the 57 samples collected from this location, Al, As, Cd, Cr, Cu, Fe, Mn, Ni, Pb, Sn, Ti and Zn concentrations were examined. Yet, regardless of the reasonable quantities of these elements, the bioconcentration levels versus the metal concentration in seawater were quite high. All the sites in this location showed considerably higher As concentrations in oral arm tissues than umbrella tissues because values were up to 4- to 2-fold higher in oral arms. At all the locations, oral arm tissue contained larger mean amounts of Fe, Zn, As, Mn and Ti than umbrella tissue, and considerable variations were noted depending on the sampling area. No notable distribution patterns were detected for Ni, Cu and Sn accumulation in jellyfish tissue.

The umbrella product’s total solids (DW) have been reported by Raposo et al. [12], which these authors compared with raw *C. tagi* umbrella (Portugal). Eleven of the twenty-five elements (Al, B, C, Fe, H, K, Mg, Mn, N, Na and P) examined in cooked umbrella displayed considerable mass variation (*p* < 0.1) versus total solids in raw umbrella. Elements Ca, Cd, Cr, Cu, I, Ni, S and Zn did not significantly differ from one another. Al was present less in cooked umbrella than in raw umbrella, and boiling eliminated the presence of As, Co, Hg, Mo, Pb, Se and V.

Three other studies [71,72,73] that examined Al content in the jellyfish often eaten by people were carried out in three cities in China. Ma et al. [71] studied residents in the Tianjin metropolis for 6 years from 2010 to 2015, and determined the risk of being exposed to dietary Al. During their research, 21.14% of the food samples contained more Al residue than that recommended (100 mg/kg). In food, the smallest mean amounts of Al were reported in 2010, with the highest levels being discovered in 2015. Jellyfish contained the largest amounts (433.28 ± 402.11 mg/kg), whereas the other foods used to feed aquatic animals obtained the lowest values (2.26 ± 5.58 mg/kg). Despite a new guideline having been established for employing Al food additives in this metropolis, this finding was probably related to its production method.

Average Al exposure in diet is 1.15 mg/kg body weight/week (bw/week). According to Zhang et al. [72], this is lower than the 2 mg/kg bw/week provisional tolerable weekly intake. That work, however, reports that jellyfish are the largest source of Al, accounting for 37.6% of daily consumption and averaging 4862 mg per kg of product.

Evaluating the degree of dietary Al consumption in Shenzhen residents (China) was the goal of research by Yang et al. [73]. In 3 days of food records, the diets of 853 people were examined. To test Al content, 1399 food samples from markets were obtained. High Al levels appeared in jellyfish from within the 318.3 to 1000.4 mg/kg range (527.5 mg/kg being the median). Children presented the greatest Al intake, and the 0–2- and 3–13-year-olds obtained exposure levels of 3.356 mg/kg bw/week and 3.248 mg/kg bw/week, respectively. These exposure levels are above the permitted threshold.

Chemical risk studies emphasize that a rigorous previous evaluation is required because of jellyfish capturing and breeding sites. The marine species herein discussed are prone to the bioaccumulation phenomenon, a process by which hazardous pollutants accumulate inside organisms and amount to more than those present in their surroundings [12,69,70]. Because of this, thorough environmental investigation is crucial before marketing jellyfish to look for potential marine contaminants like heavy metals, hydrocarbons and pesticides. This would imply the ideal option being to collect jellyfish at high seas far away from estuaries or populated regions.

Al toxicity is widely established in research into heavy metals [74]. Additionally, there are connections between Al and the onset of anemia, metabolic bone disease, neurodegenerative disorders and even genotoxic activity [75,76]. For instance, Al accumulation in the brain is able to intensify inflammatory and oxidative processes, which cause tissue damage and are a major contributor to Alzheimer’s disease etiology [77,78].

Another important aspect is related to jellyfish production and disregarding laws [79] that limit using chemical additives in food. A study on inorganic processed jellyfish components revealed that Al percentages were higher in end products than in raw ones, which highlights how the widespread usage of alum in the processing method poses a potential health hazard [80].

Al is a currently employed food additive. It comes as sodium aluminum phosphate (E 541) and Al sulfates (E 520–523) [81]. The Joint FAO/WHO Expert Committee on Food Additives (JECFA) review sets the limit for provisional tolerable weekly intake (PTWI) at 2 mg/kg bw [82]. In many European nations, the general population’s estimated daily food exposure to Al has been evaluated. Its average range lies between 0.2 and 1.5 mg/kg bw/week, and might reach 2.3 mg/kg bw/week for severely exposed consumers. Therefore, a significant population segment in Europe is expected to surpass the tolerated weekly intake (TWI), which is set at 1 mg/kg bw/week [83]. According to the “Standards for Uses of Food Additives” (GB 2760-2014) in China, 100 mg of aluminum/kg of food’s dry weight cannot be exceeded. No more than 1.8% alum is allowed in salted jellyfish according to one guideline [79].

The safety range for Al as an additive varies between Europe and China, which makes it difficult to establish a limit with absolute certainty. One work has shown that during product manufacturing, certain factors (i.e., temperature, exposure processing times and the quantity of employed alum) can affect retention and, consequently, overall Al concentrations in jellyfish tissue [14]. A research work by Ma et al. [71] revealed that the amount of Al in fish products was extremely low, unlike the elevated Al levels documented in the jellyfish taken in the same region.

Another issue could be packaging, because these items frequently lack the identifying labels required for food usage. This element has to be carefully considered given the rising number of dangers associated with the chemical makeup of packaging materials in some territories in Asia [58].

### 4.3. Allergenic Risk

Li et al. [84] report a case study about a Chinese man (26 years old) who consumed cooked salt-preserved jellyfish and had erythema, pruritus and tachycardia, dizziness and dyspnea. This man was healthy without a medical history of any allergies to drugs and/or other substances, but was violently stung by jellyfish some 6 months before. This man started noticing symptoms about 15 min after eating jellyfish. He was administered oral anti-allergic medication (10 mg/d loratadine tablets/1 week) and was given instructions about healthy diet following therapy and fluid infusion. His urticaria vanished after 5 days.

Amaral et al. [85] recruited 20 participants with bad seafood allergies and five atopic non-food allergic controls. All the patients underwent skin prick-to-prick testing (SPPT). Testing included cooked and raw umbrella, as well as challenges using *C. tagi* umbrella. Each patient negatively responded to SPPT when *C. tagi* raw umbrella was included. Neither the control participants nor the 20 patients with severe seafood allergies exhibited early- or late-phase responses to any dish. All this demonstrates that crustaceans, fish, mollusks, cephalopods and jellyfish do not react with one another.

According to another case study [86], 2 h after eating jellyfish in a salad a man (45 years old) had two anaphylactic episodes. He also had stomach cramps, palpitations, dizziness, dyspnea, chest tightness, vomiting, headaches, and even lost consciousness. PGA (polyglutamic acid) was determined as the etiological allergen for natto allergy, which began 8 years before the considered events. This man surfed, had received jellyfish stings several times, and may have also suffered anaphylactic responses after ingesting natto and jellyfish from his skin when being stung while surfing, which could have sensitized him to jellyfish nematocyte PGA. Another case concerns a 14-year-old child, who presented urticaria, coughing and dyspnea 30 min after eating a meal that included salted dried jellyfish [87]. Apart from being allergic to house dust mites, there was no previous medical history. The patient had never gone out to the sea, and had never been stung or touched by jellyfish before. The patient also had diffuse urticarial lesions, tachycardia, hypotension, edema and wheezing. To determine the implicated food, SPPT for salted and dried jellyfish was carried out. Because the patient had eaten jellyfish, anaphylactic shock was the reported cause. In a 14-year-old adolescent, wheezing and dyspnea started 1 h after a meal containing salted jellyfish. According to Wakiguchi et al. [88], anaphylaxis from eating jellyfish without PGA sensitization was the official diagnosis.

The extent of product processing (either cooked or raw) and jellyfish peptide length acting as antigens might both affect allergenic risk. According to the afore-cited research works, people with allergies to cephalopods, crustaceans, seafood, fish and mollusks can eat jellyfish without increasing the risk of allergic response [12,85]. A paper by Li et al. [84] reports an anaphylaxis incident when a subject consumed cooked and salt-preserved jellyfish. The literature contains three anaphylaxis case reports [86,87,88] after eating raw jellyfish. It would be useful to know if the jellyfish in the provided case study were properly prepared by removing all inedible portions, such as stinging tentacles and digestive and reproductive tracts. Another significant result in the literature review was lack of any cross-reactivity between jellyfish ingestion and people with an allergy to fish, seafood, crustaceans and mollusks [85].

This literature review enables us to conclude that a number of allergens can play an etiological role in anaphylaxis developing in people who have consumed meals containing jellyfish. PGA appears to be one of the allergens responsible for the development of anaphylactic responses. According to Inomata et al. [86], a man aged 45 years had two anaphylaxis episodes 2 h after consuming jellyfish salad. The discovery of raised levels of relevant IgE antibodies corroborated the subject’s natto allergy history. Furthermore, this patient reported having been regularly stung by jellyfish while surfing, which started at the age of 20. According to this paper, anaphylaxis begins when individuals, who have already been exposed to PGA from a jellyfish sting, consume fermented soy germs (high in PGA). According to two studies [85,86], as surfers tend to come into contact with jellyfish more frequently, they are more likely to experience negative responses after consuming them. This discovery could prompt those who engage in aquatic activities, and have been in contact with jellyfish, to abstain from eating such products. Inomata et al. [86] suggest that there may be a mechanism for the cross-reactivity between soy bean seeds and jellyfish. Wakiguchi et al. [88] found no evidence for this, but indicated that more allergens might induce anaphylactic response. More research is necessary to examine this and other potential relationships.

## 5. Nutritional Profile

Table 2 shows the jellyfish species’ proximate compositions as described in earlier research papers.

Jellyfish often contain a significant percentage of water (95–98% of their wet weight). Most jellyfish species have a high ash level in the dry matter, which may be related to why jellyfish live in brackish and marine waters with higher mineral contents. According to reports, desalted and ready-to-eat cannonball jellyfish (*Stomolophus meleagris* Agassiz, 1860) has a low calorie value and contains about 95% water and 4–5% proteins [14]. Some edible jellyfish species are *Acromitus hardenbergi* (Stiasny, 1934), *Rhopilema hispidum* (Vanhöffen, 1888) and *Rhopilema esculentum* (Kishinouye, 1891). They are high in minerals and protein, but low in calories and fat; according to research by Khong et al. [68] jellyfish are a recognized natural, very sustainable and low-calorie meal containing minimal calories, fat and cholesterol [21,93,94].

Anatomical jellyfish portions have different proximate compositions that are strongly impacted by the water bodies in the area [93]. Unlike gonads, which have the lowest moisture content, Doyle et al. [90] found that the umbrella of both jellyfish *Chrysaora hysoscella* (Linnaeus, 1767) and *Cyanea capillata* (Linnaeus, 1758) had a considerably higher moisture content (*p* < 0.001) than that of the oral arms. However, no significant differences appeared in the amounts of moisture in distinct *Rhizostoma octopus* (Gmelin, 1791) body sections (*p* > 0.05). Moisture content also differs across species. Based on their dry mass, edible jellyfish umbrella and oral arms have different proximate compositions that are total ash > protein > water > carbohydrate > lipid and total protein > ash > water > carbohydrate > lipid, respectively. Khong et al. [68] found that jellyfish oral arms present more protein and lipid contents than their umbrellas do. According to Costa et al. [93], no significant differences appear in jellyfish *P. noctiluca’s* proximate composition the in accordance to animal sex, except for gross energy content.

In addition to species differences, variability in jellyfish proximate compositions as shown by several works could be because of discrepancies in the analytical methods, samples and preparation techniques. By way of an example, samples were lyophilized in the works of Khong et al. [68] and Wakabayashi et al. [92], whereas Doyle et al. [90] oven-dried samples at 65 °C. Morais et al. [91] ran a proximate analysis with homogenized *Catostylus tagi* (Haeckel, 1869) jellyfish samples, but with no drying. Hsieh et al. [14] opted for ready-to-use desalted *S. meleagris*. Additionally, the majority of the research works followed AOAC methods to determine proximate composition. Khong et al. [68] determined carbohydrate content to be the difference between the non-carbohydrate component and total nutritional content, whereas Doyle et al. [90] applied a colorimetric technique to estimate carbohydrate content. Furthermore, the presenting bound water can affect the compositions’ dried jellyfish experimental results [90].

**Figure 2:**
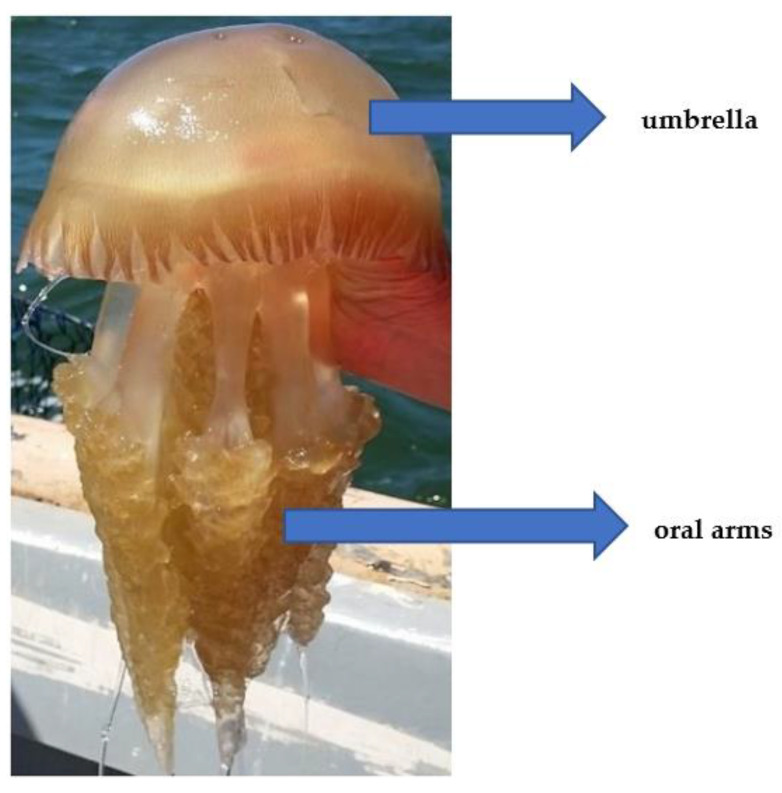
Photograph of jellyfish *Catostylus tagi* collected in the Tagus River. Edible portions (umbrella and oral arms) are pointed out.

Figure 2 illustrates a recently caught jellyfish, *C. tagi*, in Tagus River water on the Portuguese coast. Its edible portions are highlighted.

### 5.1. Energy Value

Jellyfish often have a lower calorie density than other foods, which might result from higher water and ash contents. Within a single jellyfish species, energy density changes depend on the specific tissue or body portion [68,90]. Doyle et al. [90] found that the energy densities of *C. capillata*, *R. octopus* and *C. hysoscella* umbrella tissues were significantly lower than those of the gonads and oral arms. The gonads of *R. octopus* and *C. hysoscella*, and the oral arms of *C. capillata* obtained the highest energy density. According to bomb calorimetry, the gross energy densities of the three above jellyfish species fell within the range between 2.14 ± 0.60 and 3.73 ± 0.87 kJ g DM^−1^, whereas estimates of their energy densities based on proximate compositions were between 2.83 ± 0.58 and 4.30 ± 0.75 kJ g DM^−1^ [90]. By means of bomb calorimetry, the gross energy contents of jellyfish *A. hardenbergi*, *R. hispidum* and *R. esculentum* were determined. They varied from 975.23 to 2823 kcal/kg of dry weight. Similar findings have been obtained when establishing metabolizable energy content by proximate composition with dry weights of 1194.15 ± 33.84–2624.20 ± 33.75 kcal/kg. *R. esculentum* has the most energy of the three species [68]. The energy density of jellyfish oral arms is higher than that of umbrella, according to Khong et al. [68]. Similar findings are reported by Milisenda et al. [95], who found that *P. noctiluca* gonads have a 6-fold higher energy content (11.51 J mg DW^−1^) than that of somatic tissue (2.19 J mg DW^−1^) because of their higher lipid and protein concentrations. No sex-specific difference appeared in the energy value of somatic tissue despite the much higher female gonads’ energy value (12.85 J mg DW^−1^) than that of male gonads (10.18 J mg DW^−1^). However, the jellyfish *P. noctiluca* gross energy content differed according to animal sex because the gross energy content of female umbrella was considerably higher (621 kcal 100 g^−1^) than male umbrella (357 kcal 100 g^−1^), according to the work of Costa et al. [93]. Although much lower than umbrella, the gross energy contents of female and male oral arms (respectively 151 and 174 kcal 100 g^−1^) did not statistically differ (*p* > 0.05).

### 5.2. Protein Value

According to Ding et al. [96], jellyfish are protein-rich animals. About half of all proteins in jellyfish are made up of collagen, which is the major protein. Human health may benefit from jellyfish’s higher collagen content [91]. In dried *C. capillata* and *R. octopus* jellyfish, protein makes up the majority of organic content, as Doyle et al. report [90]. The protein contents of some jellyfish species and various body tissues of one same species have been documented in several studies in the literature [68,90,91]. Jellyfish *C. tagi* [91], *A. hardenbergi*, *R. esculentum* and *R. hispidum* [68], *P. noctiluca* [93], and both the oral arms and gonads of *R. octopus* and *C. capillata* [90], all contained more protein in their oral arms than their umbrellas. The increased muscle mass density in oral arms, which facilitates mobility, might be due to their higher protein content [68]. Variations in protein levels may be caused by species, body tissue types and sample preparation/analysis procedures. Nevertheless, as Costa et al. point out [93], *P. noctiluca* protein content does not vary significantly according to sex.

The essential, conditionally essential and non-essential amino acids identified in jellyfish species respectively represent 33%, 46% and 21% of the total amino acids [68]. The total amino acids in the gonads of *R. esculentum* comprise 40.70–42.89% of essential amino acids, 47.39–50.12% of flavor amino acids and 66.55–66.92% of medicinal amino acids [97]. Leone et al. [53] state that *Aurelia* sp.1, *R. pulmo* and *C. tuberculate* respectively comprise essential amino acids at 31.4%, 50.8% and 53.6% as proportions of their total amino acid content. These results indicate likely jellyfish applications to be used as functional food and nutritional supplements.

### 5.3. Lipid Value

Jellyfish lipid content is generally low. The total lipid content found for both *R. octopus* and *C. capillata* [90], and in the umbrella tissue of *R. hispidum, R. esculentum,* and *A. hardenbergi* [68] and *Rhizostoma luteum* [25], is below 1% of their dry mass. Khong et al.’s [68] report indicates how jellyfish tissues might comprise a bigger quantity of bound fat than that of free fat because hydrolysis is vital to detect even in a small quantity of fat. Nevertheless, Leone et al. [53] report jellyfish species with higher total lipid contents that vary depending on species. Compared with *Aurelia* sp1 (4.1 ± 0.5 g/100 g DW) and *R. pulmo* (4.0 ± 0.8 g/100 g DW), *C. tuberculate* has a 3-fold higher total lipid content (12.3 ± 0.7 g/100 g DW). According to body area, the jellyfish total lipid content also varies [68,91].

Polyunsaturated fatty acids make up most of jellyfish *C. tagi’s* fatty acid composition. It is followed by saturated and monounsaturated fatty acids. Both jellyfish oral arms and umbrellas contain significantly more arachidonic acid, eicosapentaenoic acid (EPA) and docosahexaenoic acid (DHA) (about 32%) [91]. Similar results appear in the jellyfish *R. luteum* [25], where polyunsaturated fatty acids, primarily ω-3 linoleic (C18:3), essential ω-6 linoleic (C18:2) and ω-6 arachidonic (C20:4) acids, make up roughly half the fatty acid content. In jellyfish *R. pulmo* gonads, Stabili et al. [98] report that DHA and EPA and ω-3 polyunsaturated fatty acids (PUFAs) are present. However, monounsaturated and PUFAs respectively account for 15% and 14–19% of the total jellyfish *P. noctiluca* fatty acid content, which varies depending on body part, but are not based on sex [93]. Comparatively, Leone et al. [53] find that jellyfish contains about two thirds (55–70%) of saturated fatty acids, followed by polyunsaturated (25–30%) and monounsaturated (4–15%) fatty acids.

Despite total lipid content variations, jellyfish species *R. pulmo, Aurelia* sp1 and *C. tuberculate* present similar percentage compositions of fatty acids [53]. According to Wakabayashi et al. [92], *C. pacifica* and *A. aurita* have similar percentage compositions of each fatty acid.

### 5.4. Mineral Value

Na, Mg, K and Ca are the main minerals revealed in jellyfish. Morais et al. [91] noted that the *C. tagi* umbrellas and oral arms had Cl higher contents than Na. In *A. aurita, C. tagi* [91] and *C. pacifica* [92], B was the most abundant trace mineral, followed by Cu, Fe, Mn and Zn. The main elements present in jellyfish *R. esculentum, A. hardenbergi* and *R. hispidum* umbrellas and oral arms are Cl, K, Mg, Na, P, S, Si and Zn. Trace amounts of Al, As, Cu, Fe, Mo, Mn, Ni and Se also appear [68]. The seven most prevalent jellyfish *C. tagi* elements also happen to be the crucial macrominerals for human nutrition, according to Morais et al. [91].

Although Co, As, V, Mo, Se, Pb and Hg did not appear in *C. tagi* [91], tiny amounts of each mineral, except Co, have been reported in *C. pacifica* and *A. aurita* [92]. However, the entire *R. esculentum* body contains Co [99]. Only toxic metals Al and Cd appear in *C. tagi* with acceptable seafood limits [91]. Hazardous elements like Cd, Hg, Sn and Ph have been encountered within the detection limits by Khong et al. [68]. *Chrysaora fuscescens* (Brandt, 1835), *Phacellophora camtschatica* (Brandt, 1835) and *Aurelia* sp., *Chrysaora colorata* (Russell, 1964) had less Hg (0.0001–0.0006 μg/g of wet weight) and Se (0.012–0.033 μg/g of wet weight) [100].

Except for Na and K, different levels of elements in jellyfish species did not significantly differ, according to Khong et al. [68]. Wakabayashi et al. [92] report that mineral quantities and ash content in *A. aurita* were higher than in *C. pacifica*, without significantly differing amounts of trace minerals. Additionally, there was no variation in each mineral’s percentage content. *P. noctiluca* mineral composition less widely varied according to animal sex [93]. However, because Al and Zn were present in the seawater in which the *C. tagi* samples were collected this suggests that jellyfish population habitats may potentially influence mineral composition [91].

Depending on body portion, jellyfish have different mineral contents. According to several studies, jellyfish umbrella contains more ash and major minerals than oral arms and gonads [68,90,93,101]. The high concentration of major minerals in umbrella may be due to buffering processes that help the osmotic balance to be sustained. Although this promotes floating [68], oral arms have higher trace mineral levels than umbrella [91,92,93].

Unprocessed fresh jellyfish is abundant in minerals Ca, K, Mg and Na. Nevertheless, processed jellyfish products lack such minerals because of desalting and their Al concentrations are much higher than those that can be added during the alum curing process [80]. Compared with fresh jellyfish, processed jellyfish have been shown to contain detectable quantities of Cr, Ti and V, as well as much higher contents of Fe and Si. These substances might be added while processing because of impurities in tap water, curing salts and processing equipment [80,101].

## 6. Health Impacts

It is well-known that a variety of biologically active secondary metabolites is produced by marine organisms [102]. It has been suggested that jellyfish can help with weight loss, skin softening, improved digestion, and relief from conditions like back pain, ulcers, swelling, hypertension, arthritis, fatigue and exhaustion [4]. However, recent scientific studies have not adequately examined the traditional health advantages of jellyfish [50]. Furthermore, jellyfish bioactive compounds and their potential uses have been investigated less than those of distinct marine animals like sponges, microalgae and fish [26]. Jellyfish are used to develop nutraceuticals, nutricosmetics and functional foods for their high protein value and low-calorie content [68,103]. Since the 1960s, biochemical, pharmacological and toxicological research has been conducted to determine whether the active compounds of jellyfish venom can be used as medicine [104]. Because of this, several bioactive attributes of jellyfish venoms have been recently found and can be employed for medicinal purposes [105,106].

Proteinous venom can be extracted from jellyfish *R. esculentum* tentacles that performs substantial insecticidal actions against *Stephanitis pyri* (Fabricius, 1775) [107]. Apoaequorin is a jellyfish protein that has been reported to improve adults’ verbal learning [108].

The stimulatory immune effects of jellyfish *C. quinquecirrha* venom [109] and jellyfish *N. nomurai* [110] collagen extracts have been explored. Sugahara et al. [110] revealed that *N. nomurai* improves IgG and IgM production, and the generation of interferon (INF-γ), IgM in human hybridoma HB4C5 cells and tumor necrosis factor (TNF-α) in human peripheral blood lymphocytes. A novel jellyfish polysaccharide with immunomodulatory action and the potential to considerably increase RAW 264.7 macrophage cell viability has been reported by Li et al. [111]. Dendritic cells that derive from mouse bone marrow also possess proven immunostimulatory activities in response to *N. nomurai* collagen [112].

According to Ayed et al. [113], *P. noctiluca* venom and its fractions possess dose-dependent anti-inflammatory activity via NO generation inhibition in interferon gamma (IFN-)/lipopolysaccharide (LPS)-treated RAW264.7 cells. At the 12–50 g/mL concentrations, venom fractions did not, however, significantly cause cytotoxicity in RAW26.7 cells. The first anti-inflammatory fraction out of the three was able to reduce NO generation by 84%. The same study also discovered that the mRNA expression of inducible nitric oxide synthase was inhibited, which led to the transcriptional level inhibition of NO production. Hwang et al. discovered that the aqueous extracts of jellyfish *N. nomurai* had anti-inflammatory properties [114].

It has also been discovered that *R. pulmo* contains a metalloproteinase with anticoagulant activity [106]. From upside-down jellyfish *C. andromeda* venom, Mohebbi et al. [115] identified that an acetylcholine esterase inhibitory neurosteroidal alkaloid could potentially act as an effective remedy for Alzheimer’s disease. Two venom fractions and the venom of *P. noctiluca* have been shown to inhibit human plasma butyrylcholinesterase [116].

## 7. Consumer Acceptance and Sensory Analysis

Despite the nutritional and health benefits associated with jellyfish consumption, overall consumer acceptance could also be influenced by the potential risks discussed in previous sections. Jellyfish consumption has been popular for centuries in Chinese culture and in other Asian countries, but is relatively new in Europe [12]. The younger generations in the Western world are more open to try new delicacies made with jellyfish. A study conducted by the authors on the consumption of native *Catostylus tagi* jellyfish in local Portuguese cuisine confirmed that 90% of young people would accept *C. tagi* being included in their diet. They described jellyfish texture to be firm and cohesive, hydrated, quite juicy with slight adhesiveness toward suitable chewing. In addition, overall liking to indicate the acceptance level of an umbrella pâté snack revealed that the allergic volunteers showed a preference for snacks at higher jellyfish concentrations (15% and 25% supplementation levels) [12].

A similar sensory evaluation of jellyfish products was conducted at Auburn University, Alabama, USA. It included 16 inexperienced panelists who had never eaten jellyfish and 35 experienced panelists who had eaten this food. It compared the overall preference, texture and color of jellyfish cannonball umbrella and leg products processed in the laboratory to those of a commercial Malaysian product [14]. This study worked with a structured 8-point hedonic scale to assess the overall preference, crunchiness of texture, and lightness of color of an unflavored jellyfish product. The higher the score, the crunchier the texture, the lighter the color and the more preferred the product was [14]. 

In line with the sensory scores obtained in the aforementioned study, cannonball products were refrigerated for a 1-year period. Their texture was crunchier and their color was whiter than the tested commercial product. Significant differences were obtained (*p* ≤ 0.05) for the color attribute between the Malaysian sample and cannonball products. Both the cannonball products were rated as having a lighter color than the commercial product [14]. In overall preference and texture terms, the experienced panelists found that the cannonball umbrella product was crunchier than its Malaysian counterpart. The cannonball products’ preference scores were higher. The inexperienced panelists’ results indicated no significant differences in the tested samples’ crunchiness or overall preference. 

In Asia, a jellyfish food product’s peculiar crunchiness and texture are highly appreciated organoleptic qualities, and they depend very much on alum treatment. In the conventional Asian method to gain “rubber-like” jellyfish consistency, searching for alternative metals to Al is being contemplated [66]. One example is a study that aimed to replace alum with calcium salt treatment and to use phenolic compounds such ferulic acid and rutin as additional tissues to stabilize agents. Replacement indicated the product’s improved nutraceutical features. Noteworthily, the obtained products were satisfactory in nutritional, technological and microbiological terms. The procedure is an apparently efficacious processing operation to produce semifinished jellyfish food products that confirm the quality and safety requirements of the EU regulations currently in force [54]. However, one study reports that Ca ions were unable to substitute alum in collagen cross-linking when producing the rubber-like jellyfish structure. This was attributed to not only Ca ions being unable to efficiently bring down pH as alum can, but also the metal ions’ different valence [54,66]. The investigators observed that the pH values obtained at the end of treatment fell within the 4.89–7.15 range and would, thus, prevent jellyfish tissue dissolution. These findings suggest that this parameter would not be fundamental to maintain jellyfish [54]. Despite Pedersen et al. [66] proving Ca ions unsuitable for tanning, treating jellyfish samples with Ca-salt-based brine presented increased texture after a 5-day period under each testing condition and, albeit not crunchy, consistency was somewhat gelatinous-stiff. This can be accounted for by the activity of a considerable number of collagen carbonyl groups being able to chelate Ca ions, which hardens soft tissues [66]. These authors also demonstrated that treating jellyfish tissue with Ca acetate, citrate and lactate stabilizes microbial load and modifies tissue texture.

According to a related project that worked with marinated semidried jellyfish products from underutilized *Catostylus mosaicus* species, this product’s sensory evaluation and overall acceptability were investigated in Australia. The Australian jellyfish species *Catostylus mosaicus* was found to be ideally suitable for being processed for the Asian market, as evidenced by feedback from importers from Asian countries. The end product was more opaque and yellow-colored than imported salted products, although this does not necessarily detract from jellyfish acceptability. Even though rubbery flavors were noted in salted jellyfish umbrella, it did not negatively affect its overall acceptability [117]. The salting process was beyond the scope of this project, but it is believed that rubbery flavor results from the residual alum that stems from salting. These authors recommended further assessing the salting process and the salting compound that results in the best quality being determined [117]. 

Finally, if alum can be successfully replaced without compromising the nutritional and organoleptic features of a finished product, overall jellyfish acceptability in the Western world will increase. 

## 8. Conclusions

The general utilization of jellyfish can help the blue bioeconomy owing to its nutritional and health benefits. Jellyfish can help to sustain our growing population if adequately supported by the right policies. It can also promote waste reduction and the biodiversity of marine resources from local underutilized resources being employed. As it is a novel product in Europe, it is imperative to guarantee that any food products developed from jellyfish are of good quality and accepted by consumers. In addition, harmonizing the legal status associated with jellyfish as a novel food across European member states will increase its market share. Safety issues should be foremost and the most appropriate processing and most sustainable technique will be very important considerations for the future. To make them more sustainable, and as jellyfish are aquatic resources, it is important to strike a balance between the impact of mass production of jellyfish and other aquatic fish resources and valorization along the jellyfish value chain from production to consumption.

## Figures and Tables

**Table 1:** Processed jellyfish grades.

Grade	Description
A	Top-quality; no imperfections
B	Imperfections; split into halves
C	Smaller than B
D	Smaller than C
O	Imperfections; divided into little pieces

Adapted from Shin et al. [56].

**Table 2:** Proximate jellyfish compositions. Adapted from ref. [89].

Jellyfish Species	Body Part	Moisture (%)	Ash (%)	Protein (%)	Lipid (%)	Carbohydrates (%)	Energy Content	Reference
*C. capillata*	Whole body	95.8 ± 0.2	76.8 ± 2.0 *	16.5 ± 3.05 *	0.50 ± 0.10 *	0.88 ± 0.02 *	3.73 ± 0.78 kJ g DM^−1^ (BC) *	[90]
							4.30 ± 0.75 kJ g DM^−1^ (PC) *	
*R. octopus*	Whole body	96.1 ± 0.5	83.4 ± 2.9 *	12.8 ± 2.33 *	0.32 *	0.83 *	2.47 ± 0.93 kJ g DM^−1^ (BC) *	[90]
							2.83 ± 0.58 kJ g DM^−1^ (PC) *	
*S. meleagris*	Umbrella	96.10 ± 0.06	1.25 ± 0.16	2.92 ± 0.04	<0.01	-	11.68 Cal 100^−1^	[14]
*A. hardenbergi*	Umbrella	98.40 ± 0.56	48.42 ± 0.27 *	21.38 ± 0.32 *	0.38 ± 0.16 *	17.66 *	1663.60 ± 47.47 kcal/kg (BC) *	[68]
							1595.58 ± 41.91 kcal/kg (PC) *	
*R. hispidum*	Umbrella	97.80 ± 0.36	57.15 ± 0.51 *	19.95 ± 0.71 *	0.46 ± 0.28 *	18.20 *	975.23 ± 34.65 kcal/kg (BC) *	[68]
							1194.15 ± 33.84 kcal/kg (PC) *	
*R. esculentum*	Umbrella	96.02 ± 1.44	33.22 ± 0.53 *	38.12 ± 1.07 *	0.61 ± 0.06 *	8.87 *	2113.57 ± 65.12 kcal/kg (BC) *	[68]
							2005.88 ± 28.71 kcal/kg (PC) *	
*A. hardenbergi*	Oral arms	97.93 ± 0.64	31.10 ± 1.54 *	33.69 ± 1.12 *	1.08 ± 0.20 *	6.02 *	2403.00 ± 42.12 kcal/kg (BC) *	[68]
							2172.52 ± 34.70 kcal/kg (PC) *	
*R. hispidum*	Oral arms	96.14 ± 1.02	35.78 ± 0.25 *	43.80 ± 1.25 *	1.37 ± 0.17 *	10.65 *	2004.33 ± 14.14 kcal/kg (BC) *	[68]
							2115.92 ± 17.29 kcal/kg (PC) *	
*R. esculentum*	Oral arms	95.54 ± 1.75	15.90 ± 0.47 *	53.87 ± 2.11 *	1.79 ± 0.26 *	7.7 *	2823.13 ± 30.09 kcal/kg (BC) *	[68]
							2624.20 ± 33.75 kcal/kg (PC) *	
*C. tagi*	Umbrella	-	1.88	0.18	0.02	-	-	[91]
*C. tagi*	Oral arms	-	1.82	0.43	0.05	-	-	[91]
*A. aurita*	Whole body	-	76.19 *	3.49 *	0.43 *	19.90 *	-	[92]
*C. pacifica*	Whole body	-	69.05 *	7.53 *	0.72 *	22.71 *	-	[92]

* As a dry basis percentage. BC = mean gross energy density estimates by bomb calorimetry. PC = mean gross energy density estimates by proximate composition. 1 kcal/1 Cal = 4.184 kJ. - Not determined.

## Data Availability

Not applicable.

## References

[1] Food and Agriculture Organization of the United Nations, Sustainable Food and Agriculture. http://www.fao.org/sustainability/en/.

[2] Organisation for Economic Co-operation and Development, The Future of Food. http://www.oecd.org/futures/35391719.pdf.

[3] United Nations Educational, Scientific and Cultural Organization, What is Sustainable Consumption?. http://www.unesco.org/education/tlsf/mods/theme_b/mod09.html?panel=6#top.

[4] Omori M., Nakano E. (2001). Jellyfish fisheries in southeast Asia. Hydrobiologia.

[5] Food and Agriculture Organization of the United Nations, The State of World Fisheries and Aquaculture. http://www.fao.org/3/a-i5555e.pdf.

[6] Page J.W. (2015). Characterization of bycatch in the cannonball jellyfish fishery in the coastal waters off Georgia. Mar. Coast. Fish..

[7] Brotz L., Schiariti A., López-Martínez J., Álvarez-Tello J., Peggy Hsieh Y.H., Jones R.P., Mianzan H. (2017). Jellyfish fisheries in the Americas: Origin, state of the art, and perspectives on new fishing grounds. Rev. Fish Biol. Fish..

[8] Morais Z., Schiariti A., Morandini A.C., Mariottini G.L. (2017). An interdisciplinary approach to the scyphozoans of the Atlantic Ocean. Jellyfish: Ecology, Distribution Patterns and Human Interactions.

[9] Kimura S., Miura S., Park Y.H. (1983). Collagen as the major edible component of jellyfish (Stomolophus nomural). J. Food Sci..

[10] Helmholz H., Ruhnau C., Schütt C., Prange A. (2007). Comparative study on the cell toxicity and enzy matic activity of two northern scyphozoan species *Cyanea capillata* (L.) and Cyanea lamarckii (Péron & Léslieur). Toxicon.

[11] Armani A., Giusti A., Castigliego L., Rossi A., Tinacci L., Gianfaldoni D., Guidi A. (2014). Pentaplex PCR as screening assay for jellyfish species identification in food products. J. Agric. Food Chem..

[12] Raposo A., Coimbra A., Amaral L., Gonçalves A., Morais Z. (2018). Eating jellyfish: Safety, chemical and sensory properties. J. Sci. Food Agric..

[13] Edelist D., Angel D.L., Canning-Clode J., Gueroun S.K., Aberle N., Javidpour J., Andrade C. (2021). Jellyfishing in Europe: Current Status, Knowledge Gaps, and Future Directions towards a Sustainable Practice. Sustainability.

[14] Peggy H.Y., Leong F.M., Rudloe J. (2001). Jellyfish as food. Hydrobiologia.

[15] Morais Z., Soeiro R. Medusa Extracts for Inhibition of Angiotensin Converting Enzyme (ACE) Activity [in Portuguese]. https://worldwide.espacenet.com/searchResults?ST=singleline&locale=en_EP&submitted=true&DB=&query=PT106423+&Submit=Search.

[16] Zhuang Y., Sun L., Li B. (2012). Production of the angiotensin-I-converting enzyme (ACE)-inhibitory pep tide from hydrolysates of jellyfish (Rhopilema esculentum) collagen. Food Bioprocess Technol..

[17] Masuda A., Baba T., Dohmae N., Yamamura M., Wada H., Ushida K. (2007). Mucin (qniumucin), a glycoprotein from jellyfish, and determination of its main chain structure. J. Nat. Prod..

[18] Takagaki T., Sato M., Kawake T., Baba T., Kihira K., Mochida J. (2015). Interactions between jellyfish mucin and hyaluronan in human chondrocytes. Int. J. Biol. Pharm. Res..

[19] Ang C., Liu K., Huang Y. (1999). Introduction. Asian Foods: Science and Technology.

[20] Lindgren E., Harris F., Dangour A.D., Gasparatos A., Hiramatsu M., Javadi F., Loken B., Murakami T., Scheelbeek P., Haines A. (2018). Sustainable food systems—A health perspective. Sustain. Sci..

[21] Youssef J., Keller S., Spence C. (2019). Making sustainable foods (such as jellyfish) delicious. Int. J. Gastron. Food Sci..

[22] Eur-lex.europa.eu. http://data.europa.eu/eli/reg/2015/2283/oj.

[23] Purcell J.E., Baxter E.J., Fuentes V., Allan G., Burnell G. (2013). 13-Jellyfish as products and problems of aquaculture. Advances in Aquaculture Hatchery Technology.

[24] You K., Bian Y., Ma C., Chi X., Liu Z., Zhang Y. (2016). Study on the carry capacity of edible jellyfish fishery in Liaodong Bay. J. Ocean. Univ. China.

[25] Prieto L., Enrique-Navarro A., Li Volsi R., Ortega M. (2018). The large jellyfish *Rhizostoma luteum* as sustainable a re source for antioxidant properties, nutraceutical value and biomedical applications. Mar. Drugs.

[26] Merquiol L., Romano G., Ianora A., D’Ambra I. (2019). Biotechnological applications of Scyphomedusae. Mar. Drugs.

[27] Steinberger L., Gulakhmedova T., Barkay Z., Gozin M., Richter S. (2019). Jellyfish-based plastic. Adv. Sustain. Syst..

[28] Abouna S., Gonzalez-Rizzo S., Grimonprez A., Gros O. (2015). First description of sulphur-oxidizing bacterial symbiosis in a cnidarian (Medusozoa) living in sulphidic shallow-water environments. PLoS ONE.

[29] Baxter E.J., Rodger H.D., McAllen R., Doyle T.K. (2011). Gill disorders in marine-farmed salmon: Investigating the role of hydrozoan jellyfish. Aquac. Environ. Interact..

[30] Marcos-López M., Mitchell S.O., Rodger H.D. (2016). Pathology and mortality associated with the mauve stinger jellyfish Pelagia noctiluca in farmed Atlantic salmon *Salmo salar* L.. J. Fish Dis..

[31] Herrero A., Thompson K.D., Ashby A., Rodger H.D., Dagleish M.P. (2018). Complex gill disease: An emerging syndrome in farmed Atlantic salmon (*Salmo salar* L.). J. Comp. Pathol..

[32] Helmholz H., Johnston B., Ruhnau C., Prange A. (2010). Gill cell toxicity of northern boreal scyphomedusae Cyanea capillata and Aurelia aurita measured by an in vitro cell assay. Hydrobiologia.

[33] Mitchell S.O., Rodger H.D. (2011). A review of infectious gill disease in marine salmonid fish. J. Fish Diseases.

[34] Baxter E.J., Sturt M.M., Ruane N.M., Doyle T.K., McAllen R., Harman L., Rodger H.D. (2011). Gill Damage to Atlantic Salmon (Salmo salar) caused by the Common Jellyfish (Aurelia aurita) under Experimental Challenge. PLoS ONE.

[35] Bosch-Belmar M., Milisenda G., Basso L., Doyle T.K., Leone A., Piraino S. (2020). Jellyfish impacts on marine aquaculture and fisheries. Rev. Fish. Sci. Aquac..

[36] Småge S.B., Brevik Ø.J., Frisch K., Watanabe K., Duesund H., Nylund A. (2018). Correction: Concurrent jellyfish blooms and tenacibaculosis outbreaks in Northern Norwegian Atlantic salmon (*Salmo salar*) farms. PLoS ONE.

[37] Downes J.K., Yatabe T., Marcos-Lopez M., Rodger H.D., MacCarthy E., O’Connor I., Ruane N.M. (2018). Investigation of co-infections with pathogens associated with gill disease in Atlantic salmon during an amoebic gill disease outbreak. J. Fish Dis..

[38] Ferguson H.W., Christian M.J., Delannoy S.H., Nicolson J., Sutherland D., Crumlish M. (2010). Jellyfish as vectors of bacterial disease for farmed salmon (*Salmo salar*). J. Vet. Diagn. Investig..

[39] Delannoy C.M.J., Houghton J.D.R., Fleming N.E.C., Ferguson H.W. (2011). Mauve Stingers (*Pelagia noctiluca*) as carriers of the bacterial fish pathogen *Tenacibaculum maritimum*. Aquaculture.

[40] Floerl O., Sunde L., Bloecher N. (2016). Potential environmental risks associated with biofouling management in salmon aquaculture. Aquacult. Environ. Interact..

[41] Clinton M., Kintner A.H., Delannoy C.M.J., Brierley A.S., Ferrier D.E.K. (2020). Molecular identification of potential aquaculture pathogens adherent to cnidarian zooplankton. Aquaculture.

[42] Cronin M., Cusack C., Geoghegan F., Jackson D., McGovern E., McMahon T., Silke J. (2004). Salmon Mortalities at Inver Bay and mcswyne’s bay Finfish Farms, County Donegal, Ireland, during 2003.

[43] Purcell J.E., Uye S., Lo W. (2007). Anthropogenic causes of jellyfish blooms and their direct consequences for humans: A review. Mar. Ecol. Prog. Ser..

[44] Bosch-Belmar M., Azzurro E., Pulis K., Milisenda G., Fuentes V., Yahia O.K.D., Micallef A., Deidun A., Piraino S. (2017). Jellyfish blooms perception in Mediterranean finfish aquaculture. Mar Policy.

[45] Doyle T.K., De Haas H., Cotton D., Dorschel B., Cummins V., Houghton J.D.R., Davenport J., Hays G.C. (2008). Widespread occurrence of the jellyfish *Pelagia noctiluca* in Irish coastal and shelf waters. J. Plankton Res..

[46] Howell D., Schueller A.M., Bentley J.W., Buchheister A., Chagaris D., Cieri M., Townsend H. (2021). Combining ecosystem and single-species modeling to provide ecosystem-based fisheries management advice within current management systems. Front. Mar. Sci..

[47] Dickey-Collas M., Link J.S., Snelgrove P., Roberts J.M., Anderson M.R., Kenchington E., Bundy A., Brady M.M., Shuford R.L., Townsend H. (2022). Exploring ecosystem-based management in the North Atlantic. J. Fish Biol..

[48] Bastardie F., Brown E.J., Andonegi E., Arthur R., Beukhof E., Depestele J., Döring R., Eigaard O.R., García-Barón I., Llope M. (2021). A Review Characterizing 25 Ecosystem Challenges to Be Addressed by an Ecosystem Approach to Fisheries Management in Europe. Front. Mar. Sci..

[49] FAO (2020). The State of World Fisheries and Aquaculture 2020. Sustainability in Action.

[50] Leone A., Lecci R., Milisenda G., Piraino S. (2019). Mediterranean jellyfish as novel food: Effects of thermal processing on antioxidant, phenolic, and protein contents. Eur. Food Res. Technol..

[51] FAOSTAT (Food and Agriculture Organization of the United Nations Statistics Division) (2016). FAO Annu Yearb: Fishery and Aquaculture Statistics.

[52] Brotz L., Pauly D., Zeller D. (2016). Jellyfish fisheries–a global assessment. Global Atlas of Marine Fisheries: A Critical Appraisal of Catches and Ecosystem Impacts.

[53] Leone A., Lecci R.M., Durante M., Meli F., Piraino S. (2015). The Bright Side of Gelatinous Blooms: Nutraceutical Value and Antioxidant Properties of Three Mediterranean Jellyfish (Scyphozoa). Marine Drugs.

[54] Bleve G., Ramires F.A., De Domenico S., Leone A. (2021). An alum-free jellyfish treatment for food applications. Front. Nutr..

[55] Lueyot A., Rungsardthong V., Vatanyoopaisarn S., Hutangura P., Wonganu B., Wongsa-Ngasri P., Charoenlappanit S., Roytrakul S., Thumthanaruk B. (2021). Influence of collagen and some proteins on gel properties of jellyfish gelatin. PLoS ONE.

[56] Shin C., James T. (2020). Collins and Mohamad Munawar Azmi. The Technique of Edible Jellyfish Processing in Sarawak, Malaysia. Int. J. Adv. Res. Eng. Technol..

[57] Lim B.T. (1992). Malaysian Agricultural Research and Development Institute (MARDI). Res. J..

[58] Bonaccorsi G., Garamella G., Cavallo G., Lorini C. (2020). A Systematic Review of Risk Assessment Associated with Jellyfish Consumption as a Potential Novel Food. Foods.

[59] Albaraccin W., Ivan C., Raul G., Jose M. (2010). Salt in food processing, usage, and reduction—a review. Int. J. Food Sci. Technol..

[60] Rumpet R. (1991). Some Aspects of The Biology and Fishery of Jellyfish Found Along the Coast of Sarawak.

[61] Muangrod P., Rungsardthong V., Vatanyoopaisarn S., Tamaki Y., Kuraya E., Thumthanaruk B. (2021). Effect of wash cycle on physical and chemical properties of rehydrated jellyfish by-products and jellyfish protein powder. Sci. Eng. Health Stud..

[62] Charoenchokpanich W., Rungsardthong V., Vatanyoopaisarn S., Thumthanaruk B., Tamaki Y. (2020). Salt reduction in salted jellyfish (*Lobonema smithii*) using a mechanical washing machine. Sci. Eng. Health Stud..

[63] Buehler M.J. (2006). Nature designs tough collagen: Explaining the nanostructure of collagen fibrils. Proc. Natl. Acad. Sci. USA.

[64] Gambini C., Abou B., Ponton A., Cornelissen A.J. (2012). Micro-and macrorheology of jellyfish extracellular matrix. Biophys. J..

[65] Tanaka T., Fillmore D., Sun S.T., Nishio I., Swislow G., Shah A. (1980). Phase transitions in ionic gels. Phys. Rev. Lett..

[66] Pedersen M.T., Brewer J.R., Duelund L., Hansen P.L. (2017). On the gastrophysics of jellyfish preparation. Int. J. Gastron. Food Sci..

[67] Bleve G., Ramires F.A., Gallo A., Leone A. (2019). Identification of safety and quality parameters for preparation of jellyfish based novel food products. Foods.

[68] Khong N.M., Yusoff F.M., Jamilah B., Basri M., Maznah I., Chan K.W., Nishikawa J. (2016). Nutritional composition and total collagen content of three commercially important edible jellyfish. Food Chem..

[69] Epstein H.E., Templeman M.A., Kingsford M.J. (2016). Fine-scale detection of pollutants by a benthic marine jellyfish. Mar. Pollut. Bull..

[70] Muñoz-Vera A., Castejón JM P., García G. (2016). Patterns of trace element bioaccumulation in jellyfish Rhizostoma pulmo (Cnidaria, Scyphozoa) in a Mediterranean coastal lagoon from SE Spain. Mar. Pollut. Bull..

[71] Ma J., Jiang G., Zheng W., Zhang M. (2019). A longitudinal assessment of aluminum contents in foodstuffs and aluminum intake of residents in Tianjin metropolis. Food Sci. Nutr..

[72] Zhang H., Tang J., Huang L., Shen X., Zhang R., Chen J. (2016). Aluminium in food and daily dietary intake assessment from 15 food groups in Zhejiang Province, China. Food Addit. Contam. Part B.

[73] Yang M., Jiang L., Huang H., Zeng S., Qiu F., Yu M., Wei S. (2014). Dietary exposure to aluminium and health risk assessment in the residents of Shenzhen, China. PLoS ONE.

[74] Jaishankar M., Tseten T., Anbalagan N., Mathew B.B., Beeregowda K.N. (2014). Toxicity, mechanism and health e_ects of some heavy metals. Interdiscip. Toxicol..

[75] Rondeau V., Jacqmin-Gadda H., Commenges D., Helmer C., Dartigues J.F. (2009). Aluminum and silica in drinking water and the risk of Alzheimer’s disease or cognitive decline: Findings from 15-year follow-up of the PAQUID cohort. Am. J. Epidemiol..

[76] Krewski D., Yokel R.A., Nieboer E., Borchelt D., Cohen J., Harry J., Kacew S., Lindsay J., Mahfouz A.M., Rondeau V. (2007). Human health risk assessment for aluminium, aluminium oxide, and aluminium hydroxide. J. Toxicol. Environ. Health B Crit. Rev..

[77] Campbell A. (2002). The potential role of aluminium in Alzheimer’s disease. Nephrol. Dial. Transpl..

[78] Gupta V.B., Anitha S., Hegde M.L., Zecca L., Garruto R.M., Ravid R., Shankar S.K., Stein R., Shanmugavelu P., Jagannatha Rao K.S. (2005). Aluminium in Alzheimer’s disease: Are we still at a crossroad?. Cell Mol. Life Sci..

[79] Cfs.gov.hk. https://www.cfs.gov.hk/english/programme/programme_rafs/files/RA35_Aluminium_in_Food_e.pdf.

[80] Hsieh Y.P., Leong F., Barnes K.W. (1996). Inorganic constituents in fresh and processed cannonball jellyfish (*Stomolophus meleagris*). J. Agric. Food Chem..

[81] Younes M., Aggett P., Aguilar F., Crebelli R., Dusemund B., Filipič M., Frutos M.J., Galtier P., Gott D., EFSA ANS Panel (EFSA Panel on Food Additives and Nutrient Sources added to Food) (2018). Scientific Opinion on the re-evaluation of aluminium sulphates (E 520–523) and sodium aluminium phosphate (E 541) as food additives. EFSA J..

[82] World Health Organization, Food and Agriculture Organization of the United Nations (2011). Joint FAO/WHO Expert Committee on Food Additives. Evaluation of Certain Food Additives and Contaminants: Seventy-Fourth Report of the Joint FAO/WHO Expert Committee on Food Additives.

[83] European Food Safety Authority (2011). Statement of EFSA on the Evaluation of a new study related to the bioavailability of aluminium in food. EFSA.

[84] Li Z., Tan X., Yu B., Zhao R. (2017). Allergic shock caused by ingestion of cooked jellyfish: A case report. Medicine.

[85] Amaral L., Raposo A., Morais Z., Coimbra A. (2018). Jellyfish ingestion was safe for patients with crustaceans, cephalopods, and fish allergy. Asia Pac. Allergy.

[86] Inomata N., Chin K., Aihara M. (2014). Anaphylaxis caused by ingesting jellyfish in a subject with fermented soybean allergy: Possibility of epicutaneous sensitization to poly-gamma-glutamic acid by jellyfish stings. J. Dermatol..

[87] Okubo Y., Yoshida K., Furukawa M., Sasaki M., Sakakibara H., Terakawa T., Akasawa A. (2015). Anaphylactic shock after the ingestion of jellyfish without a history of jellyfish contact or sting. Eur. J. Dermatol..

[88] Wakiguchi H., Abe N., Hasegawa S. (2018). Lobonemoides robustus (jellyfish) anaphylaxis without poly––glutamic acid sensitization. Eur. J. Dermatol..

[89] Ranasinghe R.A.S.N., Wijesekara W.L.I., Perera P.R.D., Senanayake S.A., Pathmalal M.M., Marapana R.A.U.J. (2022). Nutritional Value and Potential Applications of Jellyfish. J. Aquat. Food Prod. Technol..

[90] Doyle T.K., Houghton J.D., McDevitt R., Davenport J., Hays G.C. (2007). The energy density of jellyfish: Estimates from bomb-calorimetry and proximate-composition. J. Exp. Mar. Biol. Ecol..

[91] Morais Z.B., Pintão A.M., Costa I.M., Calejo M.T., Bandarra N.M., Abreu P. (2009). Composition and in vitro antioxidant effects of jellyfish Catostylus tagi from Sado Estuary (SW Portugal). J. Aquat. Food Prod. Technol..

[92] Wakabayashi K., Sato H., Yoshie-Stark Y., Ogushi M., Tanaka Y. (2016). Differences in the biochemical compositions of two dietary jellyfish species and their effects on the growth and survival of I bacus novemdentatus phyllosomas. Aquac. Nutr..

[93] Costa R., Capillo G., Albergamo A., Li Volsi R., Bartolomeo G., Bua G., Spanò N. (2019). A multi-screening Evaluation of the Nutritional and Nutraceutical Potential of the Mediterranean Jellyfish Pelagia noctiluca. Marine Drugs.

[94] Hsieh Y.P., Rudloe J. (1994). Potential of utilizing jellyfish as food in Western countries. Trends Food Sci. Technol..

[95] Milisenda G., Rosa S., Fuentes V.L., Boero F., Guglielmo L., Purcell J.E., Piraino S. (2014). Jellyfish as prey: Frequency of predation and selective foraging of Boops boops (Vertebrata, Actinopterygii) on the mauve stinger Pelagia noctiluca (Cnidaria, Scyphozoa). PLoS ONE.

[96] Ding J.F., Li Y.Y., Xu J.J., Su X.R., Gao X., Yue F.P. (2011). Study on effect of jellyfish collagen hydrolysate on anti-fatigue and anti-oxidation. Food Hydrocoll..

[97] Yu H., Li R., Liu S., Xing R.E., Chen X., Li P. (2014). Amino acid composition and nutritional quality of gonad from jellyfish Rhopilema esculentum. Biomed. Prev. Nutr..

[98] Stabili L., Rizzo L., Fanizzi F.P., Angilè F., Del Coco L., Girelli C.R., Lomartire S., Piraino S., Basso L. (2019). The Jellyfish *Rhizostoma pulmo* (Cnidaria): Biochemical Composition of Ovaries and Antibacterial Lysozyme-like Activity of the Oocyte Lysate. Mar. Drugs.

[99] Liu X., Zhang M., Jia A., Zhang Y., Zhu H., Zhang C., Liu C. (2013). Purification and characterization of angiotensin I converting enzyme inhibitory peptides from jellyfish Rhopilema esculentum. Food Res. Int..

[100] Perrault J.R. (2019). Mercury and selenium concentrations in Scyphozoan jellyfishes and pyrosomes from Monterey Bay National Marine Sanctuary. Mar. Pollut. Bull..

[101] Liu X., Guo L., Yu H., Li P. (2012). Mineral composition of fresh and cured jellyfish. Food Anal. Methods.

[102] Krishnan S., Pachiappan P., Mazharbasha T.A., Ranganathan K. (2014). Anticancer Activity of Jellyfish, Chrysaora Quinquecirrha (Desor 1848) from Vellar Estuary, Southeast Coast of India. World J. Pharm. Res..

[103] Yusuf S., Fahmid I.M., Abdullah N. (2018). Indonesian Jellyfish as Potential for Raw Materials of Food and Drug. IOP Conf. Ser. Earth Environ. Sci..

[104] Li C., Yu H., Liu S., Xing R., Guo Z., Li P. (2005). Factors affecting the protease activity of venom from jellyfish Rhopilema esculentum Kishinouye. Bioorganic Med. Chem. Lett..

[105] Killi N., Bonello G., Mariottini G.L., Pardini P., Pozzolini M., Cengiz S. (2020). Nematocyst types and venom effects of Aurelia aurita and Velella velella from the Mediterranean sea. Toxicon.

[106] Rastogi A., Sarkar A., Chakrabarty D. (2017). Partial purification and identification of a metalloproteinase with anticoagulant activity from Rhizostoma pulmo (Barrel Jellyfish). Toxicon.

[107] Yu H., Liu X., Dong X., Li C., Xing R., Liu S., Li P. (2005). Insecticidal activity of proteinous venom from tentacle of jellyfish Rhopilema esculentum Kishinouye. Bioorganic Med. Chem. Lett..

[108] Morrill G.A., Kostellow A.B., Gupta R.K. (2017). Computational comparison of a calcium-dependent jellyfish protein (apoaequorin) and calmodulin-cholesterol in short-term memory maintenance. Neurosci. Lett..

[109] Krishnan S.U.G.A.N.T.H.I., Pachippan P.E.R.U.M.A.L. (2013). Imunomodulatory effects of the jelly fish venom C. quinquecirrha from vellar estuary, southeast coast of India. Int. J. Pharm. Pharm. Sci..

[110] Sugahara T., Ueno M., Goto Y., Shiraishi R., Doi M., Akiyama K., Yamauchi S. (2006). Immunostimulation effect of jellyfish collagen. Biosci. Biotechnol. Biochem..

[111] Li Q.M., Wang J.F., Zha X.Q., Pan L.H., Zhang H.L., Luo J.P. (2017). Structural characterization and immunomodulatory activity of a new polysaccharide from jellyfish. Carbohydr. Polym..

[112] Putra A.B.N., Nishi K., Shiraishi R., Doi M., Sugahara T. (2015). Jellyfish Collagen Stimulates Maturation of Mouse Bone Marrow-derived Dendritic Cells. J. Funct. Foods.

[113] Ayed Y., Sghaier R.M., Laouini D., Bacha H. (2016). Evaluation of anti-proliferative and anti-inflammatory activities of Pelagia noctiluca venom in Lipopolysaccharide/Interferon-γ stimulated RAW264. 7 macrophages. Biomed. Pharmacother..

[114] Hwang S.J., Ahn E.Y., Park Y., Lee H.J. (2018). An aqueous extract of Nomura’s jellyfish ameliorates inflammatory responses in lipopolysaccharide-stimulated RAW264. 7 cells and a zebrafish model of inflammation. Biomed. Pharmacother..

[115] Mohebbi G., Nabipour I., Vazirizadeh A., Vatanpour H., Farrokhnia M., Maryamabadi A., Bargahi A. (2018). Acetylcholinesterase inhibitory activity of a neurosteroidal alkaloid from the upside-down jellyfish Cassiopea andromeda venom. Rev. Bras. De Farmacogn..

[116] Ayed Y., Dellai A., Mansour H.B., Bacha H., Abid S. (2012). Analgesic and antibutyrylcholinestrasic activities of the venom prepared from the Mediterranean jellyfish Pelagia noctiluca (Forsskal, 1775). Ann. Clin. Microbiol. Antimicrob..

[117] Poole S., Naidoo R.J., Edwards J. (2018). Creating a Shelf Stable Marinated Jelly-Fish Product from the Underutilised Species Catostylus mosaicus. http://frdc.com.au/Archived-Reports/FRDC%20Projects/1998-417-DLD.pdf.

